# *N*′-Hy­droxy­pyridine-2-carboximidamide

**DOI:** 10.1107/S1600536813017418

**Published:** 2013-06-29

**Authors:** P. A. Suchetan, S. Sreenivasa, B. S. Palakshamurthy, T. Madhu Chakrapani Rao, Vijithkumar

**Affiliations:** a Department of Studies and Research in Chemistry, U.C.S, Tumkur University, Tumkur, Karnataka 572 103, India; b Department of Studies and Research in Chemistry, Tumkur University, Tumkur, Karnataka 572 103, India; c Department of Studies and Research in Physics, U.C.S, Tumkur University, Tumkur, Karnataka 572 103, India; d Tadimety Aromatics Pvt Ltd, Hirehally Industrial Area, Tumkur, Karnataka 572 168, India; e Soild State and Structural Chemistry Unit, Indian Institute of Science, Bangalore, Karnataka 560 012, India

## Abstract

The title mol­ecule, C_6_H_7_N_3_O, is almost planar (r.m.s. deviation = 0.0068 Å) and adopts an *E* conformation about the C=N double bond. In the crystal, mol­ecules are linked by pairs of strong N—H⋯N hydrogen bonds, forming inversion dimers with *R*_2_^2^(10) motifs. The dimers are further linked into *C*(3) chains through O—H⋯N hydrogen bonds.

## Related literature

For the pharmaceutical and biological activity of substituted *N*′-hy­droxy­benzamidines and 1,2,4-oxa­diazole derivatives, see: Kundu *et al.* (2012 ▶); Sakamoto *et al.* (2007 ▶); Tyrkov & Sukhenko (2004 ▶). For a related structure, see: Sreenivasa *et al.* (2012 ▶)
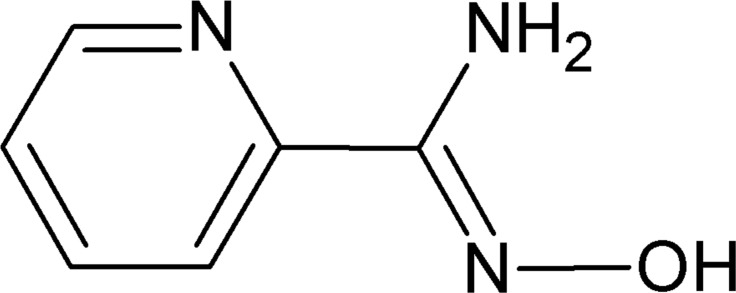


## Experimental

### 

#### Crystal data

C_6_H_7_N_3_O
*M* *_r_* = 137.15
Monoclinic, 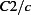

*a* = 21.367 (5) Å
*b* = 4.6382 (11) Å
*c* = 13.003 (3) Å
β = 105.468 (12)°
*V* = 1242.0 (5) Å^3^
*Z* = 8
Mo *K*α radiation
μ = 0.11 mm^−1^
*T* = 293 K
0.33 × 0.25 × 0.20 mm

#### Data collection

Bruker APEXII CCD area-detector diffractometer
Absorption correction: multi-scan (*SADABS*; Bruker, 2009 ▶) *T*_min_ = 0.966, *T*_max_ = 0.979
8242 measured reflections
1086 independent reflections
982 reflections with *I* > 2σ(*I*)
*R*_int_ = 0.038

#### Refinement

*R*[*F* ^2^ > 2σ(*F* ^2^)] = 0.031
*wR*(*F* ^2^) = 0.081
*S* = 1.08
1086 reflections
103 parameters
2 restraints
H atoms treated by a mixture of independent and constrained refinement
Δρ_max_ = 0.19 e Å^−3^
Δρ_min_ = −0.18 e Å^−3^

### 

Data collection: *APEX2* (Bruker, 2009 ▶); cell refinement: *APEX2* and *SAINT-Plus* (Bruker, 2009 ▶); data reduction: *SAINT-Plus* and *XPREP* (Bruker, 2009 ▶); program(s) used to solve structure: *SHELXS97* (Sheldrick, 2008 ▶); program(s) used to refine structure: *SHELXL97* (Sheldrick, 2008 ▶); molecular graphics: *Mercury* (Macrae *et al.*, 2008 ▶); software used to prepare material for publication: *SHELXL97*.

## Supplementary Material

Crystal structure: contains datablock(s) I, global. DOI: 10.1107/S1600536813017418/bt6916sup1.cif

Structure factors: contains datablock(s) I. DOI: 10.1107/S1600536813017418/bt6916Isup2.hkl

Supplementary material file. DOI: 10.1107/S1600536813017418/bt6916Isup3.cml

Additional supplementary materials:  crystallographic information; 3D view; checkCIF report

## Figures and Tables

**Table 1:** Hydrogen-bond geometry (Å, °)

*D*—H⋯*A*	*D*—H	H⋯*A*	*D*⋯*A*	*D*—H⋯*A*
O1—H1⋯N3^i^	0.903 (18)	1.859 (19)	2.7537 (14)	170.5 (17)
N2—H2*N*2⋯N1^ii^	0.86 (1)	2.44 (1)	3.1753 (16)	144 (1)

Symmetry codes: (i) 
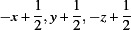
; (ii) 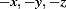.

**Fig. 1.**
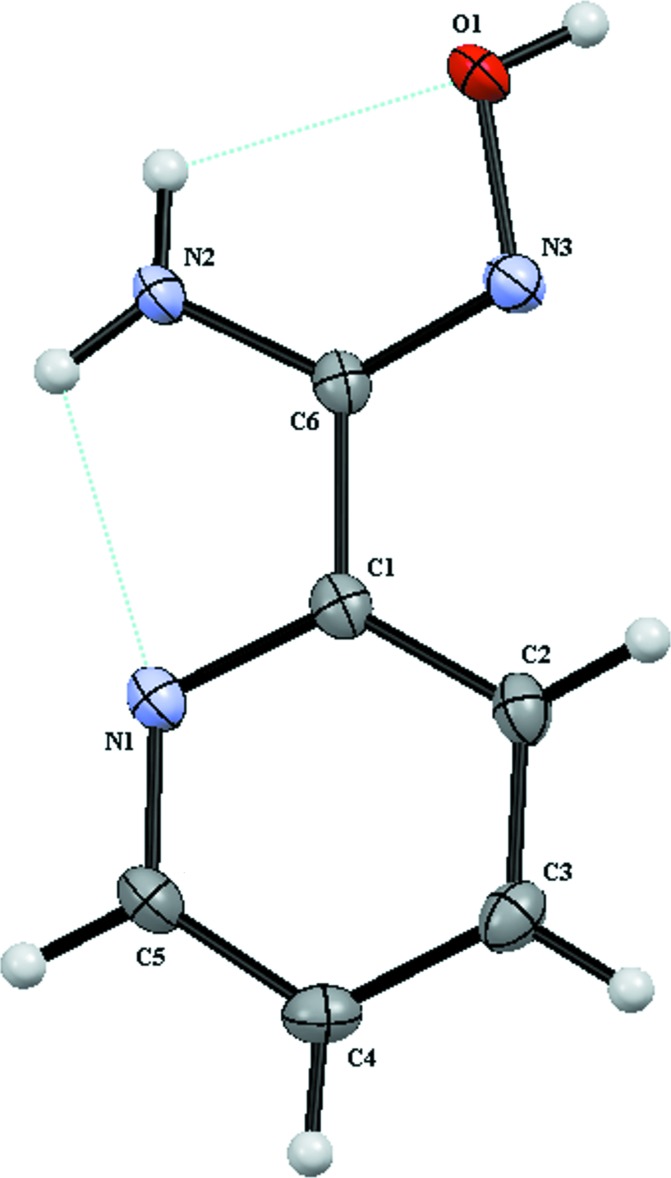
Molecular structure of the title compound, showing the atom-labeling scheme. Displacement ellipsoids are drawn at the 50% probability level.

**Fig. 2.**
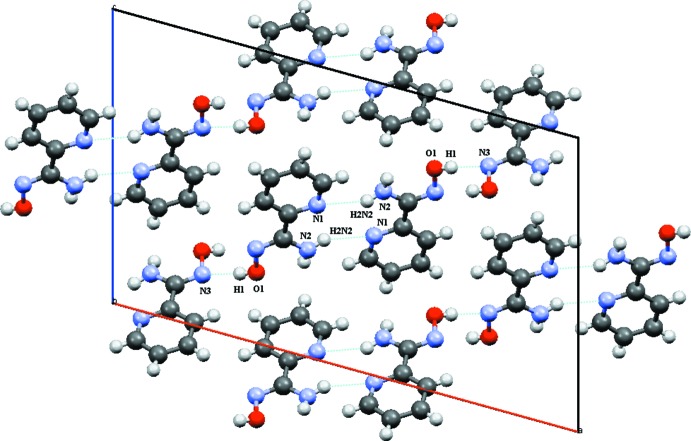
Packing of molecules displaying R22(10) loops and C(3) chains.

## supplementary crystallographic information

### Comment

Substituted *N*'-hydroxybenzamidines are important intermediates obtained during the synthesis of pharmaceutically important 1,2,4-oxadiazole derivatives (Kundu *et al.*, 2012). 1,2,4-Oxadiazole derivatives are well known for their biological activities such as anti-HIV (Sakamoto *et al.*, 2007) and anti-microbial (Tyrkov *et al.*, 2004). In this view, we synthesized the title compound to study its crystal structure.

The title compound, (I), crystalizes with a single molecule in the asymmetric unit. This is in contrast to (*E*)-3-chloro-*N*'-hydroxybenzene-1-carboximidamide, (II), (Sreenivasa *et al.*, 2012) which crystalizes with two molecules in its asymmetric unit. Compound (I) adopts an *E* configuration across the C=N double bond, as the OH group and the benzene ring are on opposite sides of the double bond, while the H atom of the hydroxy group is directed away from the NH_2_ group. This is similar as observed in (II). In the packing, the molecules are linked to one another through strong intermolecular N—H···N hydrogen bonds into *R*_2_^2^(10) motifs forming inversion dimers. The dimers are further linked into C(3) chains through O—H···N hydrogen bonds.

### Experimental

To a solution of 2-cyanopyridine (1 mmol) in ethanol was added triethyl amine (2.5 mmol) and hydroxyl amine hydrochloride, NH_2_OH.HCl (3.5 mmol). The reaction mixture was stirred at room temperature for 12hrs. (The reaction was monitored by TLC). The solvent was removed and the crude product was purified by column chromatography using hexane and ethyl acetate as the eluent.

Single crystals required for X-ray diffraction measurements were obtained from slow evaporation of the solution of the compound in a mixture of ethanol and dichloromethane (1:4).

### Refinement

The hydrogen atoms attached to N and O were located in difference maps and refined isotropically. The remaining H atoms were positioned geometrically and refined using a riding model, with C–H = 0.93 Å with isotropic displacement parameters set to 1.2 times of the *U*_eq_ of the parent atom.

### Crystal data

**Table:**

C_6_H_7_N_3_O	*F*(000) = 576
*M**_r_* = 137.15	prism
Monoclinic, *C*2/*c*	*D*_x_ = 1.467 Mg m^−^^3^
Hall symbol: -C 2yc	Mo *K*α radiation, λ = 0.71073 Å
*a* = 21.367 (5) Å	Cell parameters from 1088 reflections
*b* = 4.6382 (11) Å	θ = 2.0–25.0°
*c* = 13.003 (3) Å	µ = 0.11 mm^−^^1^
β = 105.468 (12)°	*T* = 293 K
*V* = 1242.0 (5) Å^3^	Prism, colourless
*Z* = 8	0.33 × 0.25 × 0.20 mm

### Data collection

**Table:**

Bruker APEXII CCD area-detector diffractometer	1086 independent reflections
Radiation source: fine-focus sealed tube	982 reflections with *I* > 2σ(*I*)
Graphite monochromator	*R*_int_ = 0.038
Detector resolution: 1.03 pixels mm^-1^	θ_max_ = 25.0°, θ_min_ = 2.0°
phi and ω scans	*h* = −24→24
Absorption correction: multi-scan (*SADABS*; Bruker, 2009)	*k* = −5→5
*T*_min_ = 0.966, *T*_max_ = 0.979	*l* = −15→15
8242 measured reflections	

### Refinement

**Table:**

Refinement on *F*^2^	Primary atom site location: structure-invariant direct methods
Least-squares matrix: full	Secondary atom site location: difference Fourier map
*R*[*F*^2^ > 2σ(*F*^2^)] = 0.031	Hydrogen site location: inferred from neighbouring sites
*wR*(*F*^2^) = 0.081	H atoms treated by a mixture of independent and constrained refinement
*S* = 1.08	*w* = 1/[σ^2^(*F*_o_^2^) + (0.0379*P*)^2^ + 0.8786*P*] where *P* = (*F*_o_^2^ + 2*F*_c_^2^)/3
1086 reflections	(Δ/σ)_max_ = 0.011
103 parameters	Δρ_max_ = 0.19 e Å^−^^3^
2 restraints	Δρ_min_ = −0.18 e Å^−^^3^
0 constraints	

### Special details

**Table:**

Geometry. All e.s.d.'s (except the e.s.d. in the dihedral angle between two l.s. planes) are estimated using the full covariance matrix. The cell e.s.d.'s are taken into account individually in the estimation of e.s.d.'s in distances, angles and torsion angles; correlations between e.s.d.'s in cell parameters are only used when they are defined by crystal symmetry. An approximate (isotropic) treatment of cell e.s.d.'s is used for estimating e.s.d.'s involving l.s. planes.
Refinement. Refinement of *F*^2^ against ALL reflections. The weighted *R*-factor *wR* and goodness of fit *S* are based on *F*^2^, conventional *R*-factors *R* are based on *F*, with *F* set to zero for negative *F*^2^. The threshold expression of *F*^2^ > σ(*F*^2^) is used only for calculating *R*-factors(gt) *etc*. and is not relevant to the choice of reflections for refinement. *R*-factors based on *F*^2^ are statistically about twice as large as those based on *F*, and *R*- factors based on ALL data will be even larger.

### Fractional atomic coordinates and isotropic or equivalent isotropic displacement parameters (Å^2^)

**Table:**

	*x*	*y*	*z*	*U*_iso_*/*U*_eq_	
O1	0.19134 (4)	0.1826 (2)	0.26040 (7)	0.0207 (3)	
H1N2	0.0896 (7)	0.225 (3)	0.1797 (12)	0.027 (4)*	
H2N2	0.0456 (6)	0.081 (3)	0.0861 (11)	0.027 (4)*	
H1	0.2316 (9)	0.260 (4)	0.2779 (14)	0.043 (5)*	
C1	0.12315 (6)	−0.2716 (3)	0.03188 (9)	0.0154 (3)	
C2	0.17439 (6)	−0.4236 (3)	0.01036 (10)	0.0189 (3)	
H2	0.2167	−0.3964	0.0520	0.023*	
C3	0.16144 (6)	−0.6149 (3)	−0.07361 (10)	0.0208 (3)	
H3	0.1949	−0.7164	−0.0904	0.025*	
C4	0.09770 (6)	−0.6536 (3)	−0.13259 (10)	0.0211 (3)	
H4	0.0874	−0.7834	−0.1891	0.025*	
C5	0.05004 (6)	−0.4948 (3)	−0.10538 (10)	0.0203 (3)	
H5	0.0073	−0.5229	−0.1449	0.024*	
C6	0.13390 (5)	−0.0595 (3)	0.12070 (9)	0.0147 (3)	
N1	0.06133 (5)	−0.3023 (2)	−0.02561 (8)	0.0176 (3)	
N2	0.08163 (5)	0.0824 (2)	0.13540 (9)	0.0179 (3)	
N3	0.19228 (5)	−0.0259 (2)	0.18081 (8)	0.0175 (3)	

### Atomic displacement parameters (Å^2^)

**Table:**

	*U*^11^	*U*^22^	*U*^33^	*U*^12^	*U*^13^	*U*^23^
O1	0.0156 (5)	0.0240 (5)	0.0199 (5)	−0.0027 (4)	0.0004 (4)	−0.0070 (4)
C1	0.0157 (6)	0.0151 (6)	0.0149 (6)	−0.0009 (5)	0.0032 (5)	0.0048 (5)
C2	0.0152 (6)	0.0215 (7)	0.0186 (6)	0.0008 (5)	0.0022 (5)	0.0038 (5)
C3	0.0225 (7)	0.0210 (7)	0.0205 (7)	0.0050 (5)	0.0087 (5)	0.0035 (5)
C4	0.0276 (7)	0.0199 (7)	0.0157 (6)	0.0005 (5)	0.0055 (5)	−0.0008 (5)
C5	0.0175 (6)	0.0231 (7)	0.0173 (6)	−0.0017 (5)	−0.0006 (5)	−0.0008 (5)
C6	0.0142 (6)	0.0154 (6)	0.0141 (6)	0.0000 (5)	0.0029 (5)	0.0046 (5)
N1	0.0155 (5)	0.0193 (6)	0.0162 (5)	−0.0008 (4)	0.0012 (4)	0.0004 (4)
N2	0.0130 (6)	0.0216 (6)	0.0162 (6)	0.0013 (5)	−0.0009 (5)	−0.0031 (5)
N3	0.0163 (5)	0.0177 (6)	0.0169 (5)	−0.0009 (4)	0.0018 (4)	−0.0017 (4)

### Geometric parameters (Å, º)

**Table:**

O1—N3	1.4201 (13)	C4—C5	1.3773 (18)
O1—H1	0.903 (18)	C4—H4	0.9300
C1—N1	1.3407 (16)	C5—N1	1.3408 (16)
C1—C2	1.3918 (17)	C5—H5	0.9300
C1—C6	1.4878 (17)	C6—N3	1.2928 (16)
C2—C3	1.3765 (18)	C6—N2	1.3532 (16)
C2—H2	0.9300	N2—H1N2	0.862 (13)
C3—C4	1.3849 (19)	N2—H2N2	0.859 (13)
C3—H3	0.9300		
			
N3—O1—H1	104.9 (11)	C3—C4—H4	120.9
N1—C1—C2	123.03 (11)	N1—C5—C4	124.19 (12)
N1—C1—C6	115.35 (10)	N1—C5—H5	117.9
C2—C1—C6	121.61 (11)	C4—C5—H5	117.9
C3—C2—C1	118.91 (12)	N3—C6—N2	123.75 (11)
C3—C2—H2	120.5	N3—C6—C1	118.26 (10)
C1—C2—H2	120.5	N2—C6—C1	117.97 (10)
C2—C3—C4	118.86 (12)	C1—N1—C5	116.69 (10)
C2—C3—H3	120.6	C6—N2—H1N2	116.3 (10)
C4—C3—H3	120.6	C6—N2—H2N2	119.9 (10)
C5—C4—C3	118.28 (12)	H1N2—N2—H2N2	118.9 (14)
C5—C4—H4	120.9	C6—N3—O1	108.89 (9)
			
N1—C1—C2—C3	−0.07 (18)	N1—C1—C6—N2	−0.29 (16)
C6—C1—C2—C3	−179.35 (11)	C2—C1—C6—N2	179.05 (11)
C1—C2—C3—C4	−1.19 (18)	C2—C1—N1—C5	1.51 (17)
C2—C3—C4—C5	0.96 (18)	C6—C1—N1—C5	−179.17 (10)
C3—C4—C5—N1	0.6 (2)	C4—C5—N1—C1	−1.77 (18)
N1—C1—C6—N3	178.22 (10)	N2—C6—N3—O1	−0.96 (16)
C2—C1—C6—N3	−2.45 (17)	C1—C6—N3—O1	−179.38 (9)

### Hydrogen-bond geometry (Å, º)

**Table:**

*D*—H···*A*	*D*—H	H···*A*	*D*···*A*	*D*—H···*A*
O1—H1···N3^i^	0.903 (18)	1.859 (19)	2.7537 (14)	170.5 (17)
N2—H2*N*2···N1^ii^	0.86 (1)	2.44 (1)	3.1753 (16)	144 (1)

Symmetry codes: (i) −*x*+1/2, *y*+1/2, −*z*+1/2; (ii) −*x*, −*y*, −*z*.
